# Bone marrow‐mesenchymal stem cell‐derived extracellular vesicles affect proliferation and apoptosis of leukemia cells *in vitro*

**DOI:** 10.1002/2211-5463.13352

**Published:** 2021-12-24

**Authors:** Jitrada Phetfong, Tulyapruek Tawonsawatruk, Witchayapon Kamprom, Pawared Ontong, Dalina Tanyong, Suparerk Borwornpinyo, Aungkura Supokawej

**Affiliations:** ^1^ Center for Research and Innovation Faculty of Medical Technology Mahidol University Nakhon Pathom Thailand; ^2^ Department of Orthopaedics Faculty of Medicine Ramathibodi Hospital Mahidol University Bangkok Thailand; ^3^ Department of Clinical Microbiology and Applied Technology Faculty of Medical Technology Mahidol University Nakhon Pathom Thailand; ^4^ Department of Community Medical Technology Faculty of Medical Technology Mahidol University Nakhon Pathom Thailand; ^5^ Department of Clinical Microscopy Faculty of Medical Technology Mahidol University Nakhon Pathom Thailand; ^6^ Excellent Center for Drug Discovery (ECDD) Faculty of Science Mahidol University Bangkok Thailand

**Keywords:** exosome, extracellular vesicle, leukemia, mesenchymal stem cells (MSCs), microvesicle

## Abstract

Mesenchymal stem cells (MSCs) have been proposed to have potential for tissue engineering and cell therapy due to their multilineage differentiation potential and ability to secrete numerous paracrine factors, including extracellular vesicles (EVs). Increasing evidence has demonstrated that MSC‐derived EVs (MSC‐EVs) are able to induce the repair of tissue damage and regulate the immune system. However, their role in cancer development is still unclear. Reports have suggested that whether MSC‐EVs have an inhibitory or promoting effect on cancer is dependent on the type of cancer. In this study, the role of MSC‐EVs in the regulation of leukemic cell growth *in vitro* was investigated. The EVs were collected from conditioned media of MSCs by ultrafiltration using a 10 kDa molecular weight cutoff (MWCO) filter. The isolated MSC‐EVs were comprised of microvesicles and exosomes, as examined by the size of vesicles and exosomal proteins, CD81 and flotillin‐1. Cell proliferation, cell cycle status, apoptosis, and gene expression were examined in the leukemic cell lines NB4 and K562 after treatment with MSC‐EVs. Suppression of cell proliferation and induction of apoptosis was observed. Gene expression analysis revealed differential expression of apoptotic‐related genes in NB4 and K562. MSC‐EVs increased the expression of *BID* and *BAX* and decreased expression of *BCL2*, indicating the induction of intrinsic apoptosis in NB4. In contrast, MSC‐EVs increased the expression of the death receptor gene *TRAILR2* and cell cycle regulator genes *P21* and *CCNE2* in K562. In conclusion, MSC‐EVs partially induce leukemic cell apoptosis, and thus may have potential for the development of supportive therapies for leukemia.

## Graphical abstract

This study reveals the *in vitro* inhibitory effect of extracellular vesicles (EVs) derived from bone marrow‐mesenchymal stem cells (BM‐MSCs) on leukemic cells, NB4 and K562. The EVs comprising microvesicles and exosomes partially induce leukemic cell apoptosis through activation of intrinsic and extrinsic apoptosis pathways in NB4 and K562, respectively. The MSC‐EVs may have potential for development of supportive therapies for leukemia.

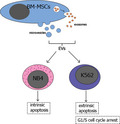

**Abbreviations**
BAX
: BCL2‐associated X apoptosis regulator
BCL2
: apoptosis regulator
BID
: BH3 interacting domain death agonist
CCNE2
: cyclin E2
FAS
: Fas cell surface death receptor
GAPDH
: glyceraldehyde‐3‐phosphate dehydrogenase
P21
: cyclin‐dependent kinase inhibitor 1A (CDKN1A)
P53
: tumor protein p53
PUMA
: p53 upregulated modulator of apoptosis
TRAILR2
: TNF‐related apoptosis‐inducing ligand receptor 2

Mesenchymal stem cells (MSCs) are multipotent stem cells that have the ability to self‐renew and differentiate into multiple cell types in the mesodermal lineage. A variety of tissue types is found to be the source of MSCs [1]. MSCs have been extensively studied during the past decade, in particular, in the field of regenerative medicine. In addition to MSC themselves, accumulating evidence reveals that MSCs exert effects on surrounding cells through the paracrine activity by secretion of various soluble factors including extracellular vesicles (EVs) [2, 3]. Generally, there are three types of EVs released from the cells―exosomes, microvesicles, and apoptotic bodies that are classified by the mechanism of biogenesis. However, the EVs can be classified based on their characteristics that are examined *in vitro* according to the Minimal Information for Studies of Extracellular Vesicles (MISEV) criteria [4]. Among the subtypes of EVs, exosomes and microvesicles have been investigated and are proposed to use as a cell‐free therapeutic approach that could overcome the limitations of cell therapy. Exosomes, the smallest type of EVs (typically 40–100 nm), are generated from the internal vesicles of multivesicular bodies (MVBs), which are subsequently released into the extracellular space, while microvesicles (50–1000 nm) are shed by outward blebbing of the plasma membrane [5]. EVs serve as a vehicle carrying a variety of molecules, including proteins, lipids, metabolites, and nucleic acid (microRNA, mRNA) to function in intercellular communication in normal physiological and also pathological conditions. In recent years, MSC‐derived EVs (MSC‐EVs) have demonstrated favorable results for the treatment of various diseases, including cancers. However, the role of MSC‐EVs in cancer is still controversial. They exhibited antitumor activity in some cancer types such as pancreatic cancer [6], prostate cancer [7], and glioma [8] while they showed promoting effects on the others [9, 10, 11]. This variation was suggested to be in accord with the type of cancer as well as the source of MSCs and genetic modification of MSCs, which need to be clarified before clinical use.

Leukemia is a result of uncontrolled proliferation of abnormal immature hematopoietic cells, which further accumulate in the bone marrow and interrupt normal hematopoiesis. There are several types of leukemia, classified by characteristics of abnormal blood cell types that respond to therapy differently. Hematopoietic stem cell transplantation (HSCT) is considered the curative treatment in postremission therapy of leukemia. The roles of MSCs in HSCT have been investigated in various conditions. Evidence revealed a favorable effect of MSCs on the prevention of graft‐versus‐host disease (GVHD) in patients with hematological malignancies undergoing allogeneic HSCT [12, 13]. Interestingly, it has been demonstrated that MSC‐EVs recapitulated the immunomodulatory roles of MSCs in the GVHD condition indicating alternative MSC products for clinical use [14, 15]. However, the role of MSC‐EVs on leukemic cells has not been clearly elucidated. Importantly, there are a limited number of previous studies that demonstrated the activity of unmodified MSC‐EVs on leukemic cells.

In the present study the effect of MSC‐EVs on leukemic cells was investigated. The MSCs were isolated from bone marrow and MSC‐EVs were collected from conditioned medium of MSC culture. The leukemic cell lines, NB4 and K562, which are derived from acute promyelocytic leukemia and chronic myeloid leukemia, respectively, were used in this study. The results revealed the inhibitory effect of MSC‐EVs on both leukemic cells, thus supporting the antitumor activity of MSC‐EVs.

## Materials and methods

### Cell culture

Bone marrow‐derived MSCs (BMMSCs) were isolated from subjects during the operation. The procedure was approved by the Ethical Committee of the Faculty of Medicine, Ramathibodi Hospital, Mahidol University (MURA2017/603). The study methodologies conformed to the standards set by the Declaration of Helsinki. Written informed consent was obtained from all subjects before sample collection. The mononuclear cells were isolated from bone marrow aspiration by density gradient centrifugation using Histopaque‐1077 (Sigma‐Aldrich, St. Louis, MO, USA). The isolated mononuclear cells were washed with phosphate‐buffered saline (PBS) and seeded into a tissue culture flask in a growth medium containing Dulbecco's modified Eagle's medium (DMEM, Gibco, Grand Island, NY, USA), 10% fetal bovine serum (FBS, Merck, Darmstadt, Germany), and 1% penicillin/streptomycin (Gibco). The cells were cultured for 3 days before removing the floating cells. The adherent cells were cultured until 80% confluence and were ready to be passaged using 0.5% trypsin‐EDTA (Gibco). Bone marrow‐derived MSCs were characterized for the typical MSC markers including surface marker expression and multilineage differentiation potency as previously described [16]. Briefly, the expression of CD73, CD90, CD105, CD34, and CD45 was assessed by flow cytometry. Osteogenic and adipogenic differentiation was determined by culturing the cells in osteogenic and adipogenic differentiation medium for 14–21 days before staining with Alizarin Red S and Oil Red O, respectively.

The leukemic cell lines, NB4 and K562, were purchased from ATCC (Rockville, MD, USA). Leukemic cells were cultured in Roswell Park Memorial Institute 1640 Medium (RPMI, Gibco) supplemented with 10% FBS and 1% penicillin/streptomycin. Cells were passaged at a ratio of 1:4 every 2 days. The cell viability was determined by trypan blue staining (Gibco) during culture.

### Isolation of EVs from conditioned medium of MSCs

**Fig. 1:**
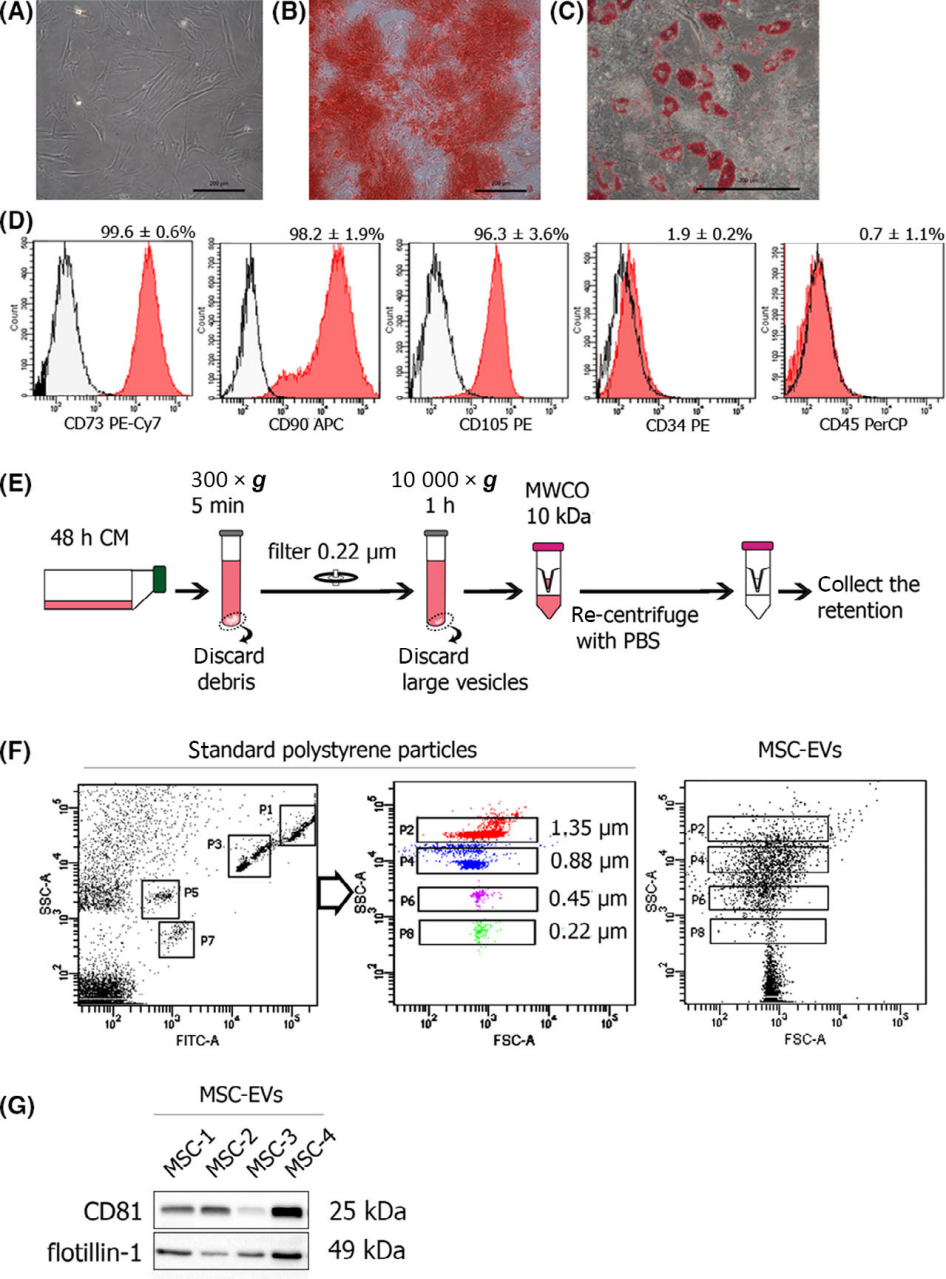
Characterization of bone marrow‐derived MSCs and MSC‐EVs. (A) The MSCs display fibroblast‐like morphology. (B) Alizarin Red S staining shows the matrix mineralization, the characteristic of osteogenic differentiation. (C) Oil Red O staining shows the differentiated adipocytes. (D) Flow cytometric analysis of cellular markers including CD73, CD90, CD105, CD34, and CD45 of the MSCs is shown. The signal from the unstained control is presented in the white histogram. The percentage of each CD marker’s positive cells are presented as mean ± SD (*n* = 4). (E) Schematic diagram shows the process of EV isolation from 48 h CM of MSCs. (F) Flow cytometric analysis shows the pattern of the standard fluorescent polystyrene particles that was gated by FITC intensity followed by the forward scatter (FSC) and side scatter (SSC) plot. The size of the MSC‐EVs was determined using FSC and SSC plots according to the standard particles. (G) Exosomal proteins, CD81 and flotillin‐1, of MSC‐EVs were examined by western blot (*n* = 4). Scale bar, 200 µm.

Bone marrow‐derived MSCs at passage 4–5 were seeded into a tissue culture flask at a density of 5 × 10^5^ cells/T75 and cultured in growth medium for 2 weeks. The medium was removed and the cells were washed several times with PBS. After discarding the PBS, 10 mL of serum‐free medium was added to the flask and the cells were cultured for 48 h before collecting the conditioned medium (CM). The CM was centrifuged at 300 × g for 5 min to remove cell debris followed by filtration with a 0.22‐µm filter. To remove apoptotic bodies, the CM was centrifuged at 10,000 × g for 1 h and the pellet was discarded. The supernatant was collected and ultrafiltration performed using 10 kDa MWCO filter (Amicon, Beverly, MA, USA) to collect the EVs. At the last step of ultrafiltration, the column was filled with PBS and recentrifuged to suspend the EVs in PBS (Fig. 1E). The EV protein was quantified using the BCA assay (Pierce, Rockford, IL). The size of the EVs was examined by flow cytometry comparing it with the standard fluorescent polystyrene particles with sizes ranging from 0.22 µm to 1.35 µm (SheroTech, Lake Forest, IL, USA).

### Western blot

Exosomal protein makers were determined using western blot. Fifteen micrograms of EVs protein were resolved with sodium dodecyl sulfate‐polyacrylamide gel electrophoresis (SDS‐PAGE, Sigma‐Aldrich) and blotted to a polyvinylidene difluoride (PVDF) membrane (Merck Millipore, Bedford, MA, USA). The blotted membrane was blocked with 5% skim milk (Sigma‐Aldrich) for 1 h at room temperature followed by staining with anti‐CD81 (1:250, cat. 10630D, Invitrogen, La Jolla, CA, USA) and antiflotillin‐1 (1:2000, cat. F1180, Sigma‐Aldrich) at 4 °C overnight. The membrane was washed with buffer several times to remove primary antibodies before staining with horseradish peroxidase (HRP)‐conjugated secondary antibody for 1 h at room temperature. The chemiluminescent signal was developed using ECL Prime western blotting detection reagent (GE Healthcare, Chicago, IL, USA) and visualized by chemiluminescent detection instrument (Bio‐Rad, Hercules, CA, USA).

### Proliferation assay

Proliferation of leukemic cell lines after treatment with MSC‐EVs was examined by MTT assay. The leukemic cell lines were seeded in triplicate at a density of 1.5 × 10^4^ cells/well of the 96‐well plate and treated with 0, 50, and 100 µg·mL^−1^ MSC‐EVs in a reduced‐serum medium (RPMI, 2% FBS, 1% penicillin/streptomycin) for 48 h. Fifty microliters of 1 mg·mL^−1^ MTT reagent (Invitrogen) was added to the well and the plate was incubated for 4 h in a humidified 37 °C incubator. To dissolve formazan crystals formed in the cells, 100 µL of 10% SDS in 0.01 M hydrochloric acid was added to the well and incubated overnight for complete dissolution. The optical density at 540 nm was measured using a spectrophotometer microplate reader. The data were collected as the absorbance value and presented as percent of control (*n* = 3).

### Apoptosis analysis

Cell apoptosis was examined using FITC Annexin V apoptosis detection kit (BD Biosciences, San Jose, CA, USA) and analyzed by flow cytometry. The leukemic cell lines were treated with 0 and 100 µg·mL^−1^ of MSC‐EVs for 48 h. The cells were collected, washed twice with cold PBS, and suspended in 1x binding buffer at a concentration of 1 × 10^6^ cell·mL^−1^. One hundred microliters of cell suspension were mixed with 5 µL of FITC‐conjugated Annexin V and 5 µL of Propidium iodide (PI). After incubation for 15 min at room temperature, 400 µL of 1x binding buffer was added to the suspension and performed flow cytometric analysis within 1 h using FACS Canto II and FACSDiva software (BD Biosciences, San Jose, CA, USA).

### Cell cycle analysis

The cell cycle was examined using PI staining and analyzed by flow cytometry. The leukemic cell lines were treated with 0 and 100 µg·mL^−1^ of MSC‐EVs for 48 h. The cells were collected, washed with cold PBS, and suspended in 1 mL of cold PBS. To fix the cells, cell suspension was added dropwise to 4.5 mL of cold 70% ethanol, mixed thoroughly, and incubated for 12–24 h at 4 °C. Cell suspension was centrifuged at 500 × ***g***, 5 min to discard the ethanol. After washing with PBS, the cell pellet was suspended in 1 mL of PI staining solution containing 10 µg·mL^−1^ PI, 100 µg·mL^−1^ RNase A, and 0.1% Triton X‐100 in PBS (all from Sigma‐Aldrich). The cells were incubated for 15 min at room temperature before flow cytometric analysis using FACS Canto II and facsdiva software (BD Biosciences).

### Gene expression

The leukemic cell lines were treated with 0, 50, and 100 µg·mL^−1^ of MSC‐EVs for 48 h. Total RNA was harvested using TRIzol reagent according to the manufacturer’s instruction (Invitrogen). The quantity and purity of RNA was examined by OD at 260 nm and the ratio of OD 260/280, respectively, using NanoDrop (ThermoFisher, Waltham, MA, USA). One microgram of RNA was reverse transcribed to cDNA using RevertAid first strand cDNA synthesis kit (Thermo Scientific, Waltham, MA, USA). Real‐time polymerase chain reaction (PCR) assay was carried out by CFX Bio‐Rad using SYBR green master mix (Kappa) and the primers specific to the interested genes (Table 1). The primer sequences were designed by a primer designing tool (NCBI, Bethesda, MD, USA), Primer 3, and BLAST. The nucleotide sequences of the genes were obtained from the NCBI database. The gene expression was normalized to glyceraldehyde‐3‐phosphate dehydrogenase (*GAPDH*, housekeeping gene) and presented as a relative level to control.

**Table 1:** Primer sequences.

Gene	Primer sequences (5’–3’)	Product size (bp)
*BID*	Forward: AGACTGATGGCAACCGCAG	133
	Reverse: GGGATGCTACGGTCCATGCT	
*BAX*	Forward: AGGATGCGTCCACCAAGAAG	137
	Reverse: AGCTGCCACTCGGAAAAAGA	
*BCL2*	Forward: TCCTGCATCTCATGCCAAGG	191
	Reverse: TCCCAGAGGAAAAGCAACGG	
*PUMA*	Forward: GGATGAAATTTGGCATGGGGT	168
	Reverse: TAAGGGCAGGAGTCCCATGA	
*FAS*	Forward: AATAAACTGCACCCGGACCC	192
	Reverse: AGAAGACAAAGCCACCCCAA	
*TRAILR2*	Forward: TAAGTCCCTGCACCACGAC	190
	Reverse: CCACTGTGCTTTGTACCTGATTC	
*P53*	Forward: CCTCTCCCCAGCCAAAGAAG	100
	Reverse: GCCTCATTCAGCTCTCGGAA	
*P21*	Forward: GATGAGTTGGGAGGAGGCAG	156
	Reverse: CTGAGAGTCTCCAGGTCCAC	
*CCNE2*	Forward: GCTGGTCTGGCGAGGTTTT	248
	Reverse: AATGCAAGGACTGATCCCCC	
*GAPDH*	Forward: CAACTACATGGTTTACATGTTCCAA	206
	Reverse: CAGCCTTCTCCATGGTGGT	

### Statistical analysis

Data are presented as mean ± standard deviation (SD). Statistical analysis was assessed using graphpad Prism v. 5.00 for Windows (graphpad software, San Diego, CA, USA). Student’s *t*‐test was used to evaluate the significant difference between two groups while the ANOVA test with Tukey's multiple comparison test was used to evaluate the difference of multiple groups. The difference was considered statistically significant at *P* < 0.05.

## Results

### Characterization of bone marrow‐derived MSCs and MSC‐EVs

After culture *in vitro*, bone marrow‐derived MSCs showed a plastic adherent property with fibroblast‐like morphology (Fig. 1A). Typical characteristics of MSCs were examined according to the minimal criteria defined by the International Society for Cellular Therapy [17]. The MSCs demonstrated trilineage differentiation potency when cultured in differentiation induction medium as shown by positive staining for Alizarin Red S, Oil Red O staining, and expression of the chondrogenic gene (Fig. 1B,C, Fig. S1). Immunophenotypic analysis revealed that more than 95% of MSCs were positive for CD73, CD90, CD105 and less than 2% were positive for CD34 and CD45 (Fig. 1D). The characterized MSCs from four donors were continuously grown for EV isolation separately. After differential centrifugation of the CM to remove apoptotic bodies that are large vesicles (>1 µm), the EVs were collected by ultrafiltration with 10 kDa MWCO filter (Fig. 1E). By this isolation technique, 1–2 mg of MSC‐EV protein was collected from 100 mL CM. The size of the isolated MSC‐EVs examined by comparison with the standard polystyrene particles was ~200 nm to 1 µm (Fig. 1F). Although the conventional flow cytometer could not distinguish the exosomes, which are smaller than 200 nm from the noise, the isolated MSC‐EVs expressed the specific exosomal proteins, CD81, and flotillin‐1 as examined by western blot (Fig. 1G). These results indicated the mixture of exosomes and microvesicles in the isolated MSC‐EVs.

### Effect of MSC‐EVs on the proliferation of leukemic cells

**Fig. 2:**
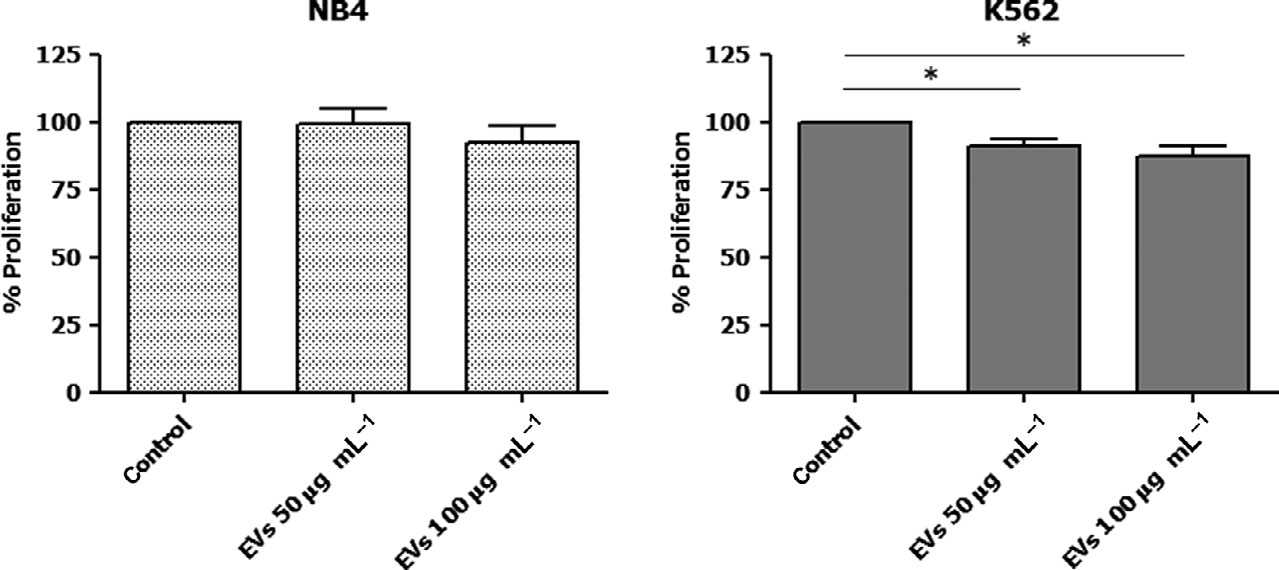
Effect of MSC‐EVs on the proliferation of leukemic cells. NB4 and K562 were treated with MSC‐EVs at concentrations of 50 and 100 µg·mL^−1^ for 48 h. The experiment was performed three times using EVs collected from three lines of MSCs (*n* = 3). Cell proliferation was examined by MTT assay. The MTT results (absorbance values) are presented as percentage to the control group of each experiment before statistical analysis (mean ± SD). *P < 0.05 versus control. Statistical significance was determined using one‐way ANOVA followed by Tukey's multiple comparison test.

The leukemic cell lines, NB4 and K562, were treated with 50 and 100 µg·mL^−1^ MSC‐EVs for 48 h before assessing cell proliferation by the MTT assay. The proliferation of NB4 was not different from control after treatment with 50 and 100 µg·mL^−1^ MSC‐EVs. In K562, treatment with 50 and 100 µg·mL^−1^ MSC‐EVs exhibited a significant decrease in the growth potential compared with control, suggesting the inhibitory effect of MSC‐EVs on the proliferation of K562 (Fig. 2).

### Effect of MSC‐EVs on cell cycle status and apoptosis of leukemic cells

**Fig. 3:**
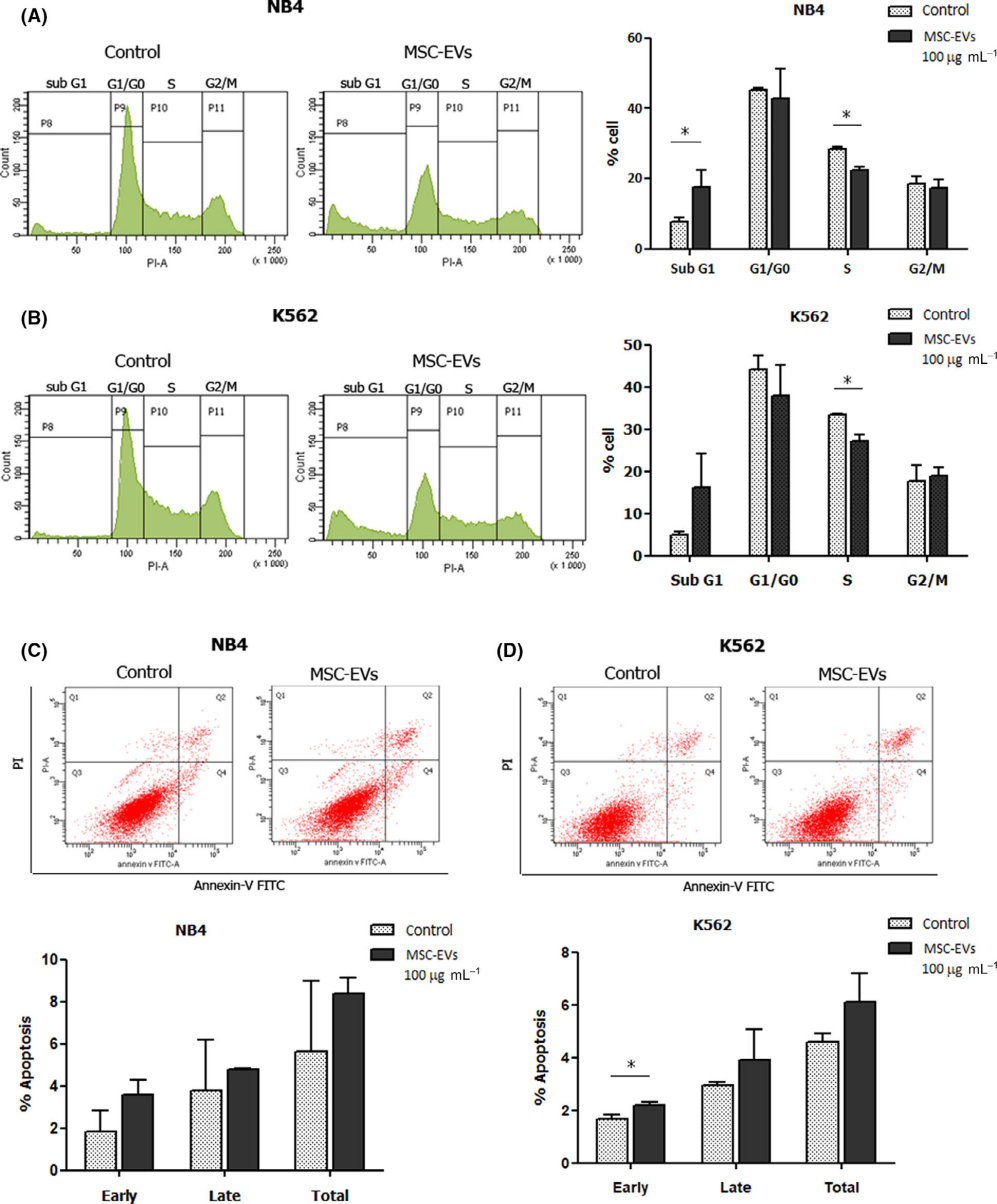
Effect of MSC‐EVs on cell cycle and apoptosis of leukemic cells. Histogram shows the cell cycle analysis according to the intensity of PI staining in (A) NB4 and (B) K562 after treatment with 100 µg·mL^−1^ MSC‐EVs for 48 h. Graphs present the percentage of the cells in each phase (*n* = 3). *P < 0.05 versus control. Flow cytometric analysis of Annexin‐V/PI staining of (C) NB4 and (D) K562 after treatment with 100 µg·mL^−1^ MSC‐EVs for 48 h. Graph presents the percentage of early (Annexin‐V+, PI‐), late (Annexin‐V+, PI+), and total (Annexin‐V+) apoptotic cells of leukemic cells (*n* = 3). *P < 0.05 versus control. Statistical significance was determined using Student’s *t*‐test.

To investigate whether the inhibitory effect of MSC‐EVs on leukemic cell proliferation involved the regulation of the cell cycle, the cell cycle phase of NB4 and K562 was examined by PI staining. Leukemic cells were treated with 100 µg·mL^−1^ MSC‐EVs for 48 h before PI staining. The results revealed that MSC‐EVs influenced the cell cycle phase of leukemic cells. Sub‐G1 population that was relevant to apoptotic cell death was found to be significantly increased, while the S phase population were decreased in NB4 after MSC‐EV treatment (Fig. 3A). In K562, MSC‐EVs significantly decreased the S phase population, while they slightly increased the sub‐G1 population; however, it was not significantly different from the control (*P* = 0.0751) (Fig. 3B). The ability of MSC‐EVs to induce leukemic cell apoptosis was examined by Annexin‐V/PI staining. In NB4, the early and late apoptosis were not different between MSC‐EVs and the control group (Fig. 3C). In K562, the early apoptosis was significantly increased after MSC‐EV treatment; however, the total apoptosis was not different from the control group (*P* = 0.0774) (Fig. 3D).

### MSC‐EVs increased the expression of apoptotic‐related genes

**Fig. 4:**
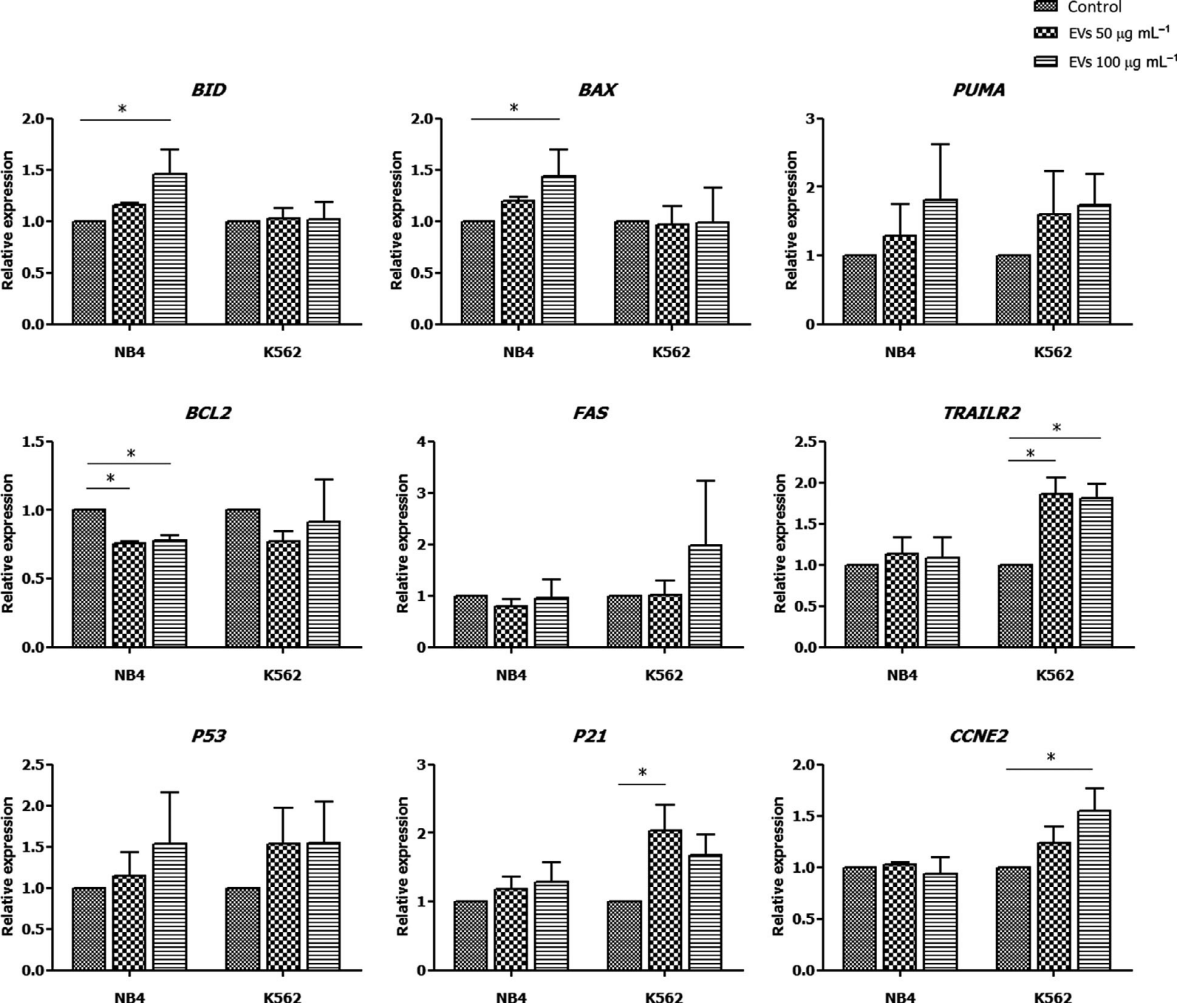
Relative expression of genes involved with apoptosis and cell cycle arrest in leukemic cell after MSC‐EV treatment. The level of mRNA expression was normalized to *GAPDH*, a housekeeping gene. The expression of each gene is presented as relative expression compared to the control group (mean ± SD). *P < 0.05 versus control (*n* = 3). Statistical significance was determined using one‐way ANOVA followed by Tukey's multiple comparison test.

The expression of genes involved with apoptosis and cell cycle arrest was examined in NB4 and K562 after MSC‐EV treatment for 48 h. In NB4, MSC‐EVs significantly increased the expression of the proapoptotic genes, *BID* and *BAX*, while it decreased the expression of the antiapoptotic gene, *BCL2,* compared with the control. In contrast, the expression of *BID*, *BAX*, and *BCL2* in K562 after MSC‐EV treatment was not different from the control. Interestingly, MSC‐EVs increased the expression of the death receptor, *TRAIL2* in K562. The *P53* and *PUMA* were likely induced; however, there were no significant differences between MSC‐EVs and the control group both in NB4 and K562. The expression of the cell cycle regulator gene, *P21,* and its downstream target gene, *CCNE2,* were significantly increased in K562 after MSC‐EV treatment, while they were not affected in NB4 (Fig 4).

## Discussion

During the last decade, MSC‐EVs have received growing interest in regenerative medicine due to their potential to regulate immune systems and stimulate tissue regeneration [18]. In cancer, the results obtained from numerous studies demonstrated a dual role of MSC‐EVs on cancer development and progression that might depend on the type and stage of the cancer as well as the source of MSCs and the methodology of EV isolation [19]. Those evidences have led to attentive consideration of using MSC‐EVs in cancer therapy. In the present study, EVs were collected from bone marrow‐derived MSCs by ultrafiltration with a 10 kDa MWCO filter that has been used as an alternative method to ultracentrifugation to reduce vesicle loss and damage [20]. The MSC‐EVs that comprised exosomes and microvesicles showed the suppressive effect on leukemic cells growth *in vitro*. Although the effect was not clearly demonstrated, the MSC‐EVs partially induced apoptosis and cell cycle arrest in NB4 and K562. These results were similar to the previous studies that revealed the antiproliferative and proapoptotic effect of MSC‐EVs on leukemic cells, although the EVs were derived from different sources and techniques [21, 22, 23]. To reveal the potential use of EVs in a cancer treatment application, the combination of MSC‐EVs and chemotherapeutic agents has been studied. The MSC‐EVs could increase the sensitivity of chemotherapeutic drugs to induce leukemic cell apoptosis suggesting the advantage of MSC‐EVs in supportive treatment, particularly in the case with chemo‐resistance [24].

Even though the cellular characteristics of NB4 and K562 after exposure to MSC‐EVs were similar, the apoptosis‐associated genes expression analysis was different between NB4 and K562. In NB4, the MSC‐EVs increased the expression of the proapoptotic genes, *BID* and *BAX*, while it decreased the expression of the antiapoptotic gene, *BCL2*, indicating the induction of the intrinsic apoptosis pathway. The MSC‐EVs rather induced extrinsic apoptosis in K562 through increasing the expression of *TRAILR2*, the death receptor. Furthermore, MSC‐EVs increased the expression of *P21* and its downstream target gene, *CCNE2*, in K562, indicating the induction of cell cycle arrest in K562. P53 has been identified to play a central role in regulating apoptosis and cell cycle arrest. P53 has diverse roles in modulating apoptotic pathways by direct induction of several proapoptotic genes in the BCL2 family (*PUMA*, *NOXA*, *BID*, *BAD*, *BAX*, *BAK*) and death receptor genes (*FAS*, *TRAILR*) that lead to intrinsic and extrinsic apoptosis, respectively. In addition, p53 negatively controls cell cycle by inducing cyclin‐dependent kinase inhibitor (p21) resulting in G1/S cell cycle arrest [25]. In the present study, several target genes of p53 were found to be activated in NB4 and K562 after MSC‐EV treatment, suggesting that the apoptosis might be mediated, at least in part, by the p53‐mediated pathway. However, the expression of the *P53* gene was not increased by the MSC‐EVs, suggesting that there might be other pathways involved, which needs further study. Various signaling molecules and microRNAs (miRNAs) have been investigated to be involved in the antitumor activity of MSC‐EVs. Most of the studies focused on the function of miRNAs carried by the MSC‐EVs. The study of miRNA profiling revealed a diversity of miRNAs in MSC‐EVs, some of which have been reported to play roles in cancer‐regulatory activity by targeting several cancer‐survival pathways [26]. Additionally, genetically modified MSC‐EVs with overexpression of certain miRNAs demonstrated a beneficial effect in antitumor activity, while unmodified MSC‐EVs showed the variation of results [27]. This evidence implies that to use MSC‐EVs as an effective cancer therapeutic tool, genetic modification of MSC‐EVs is required.

Owing to the controversial effects of MSC‐EVs on cancer cells, the use of MSC‐EVs in cancer therapy has to be considered. As reported here, although MSC‐EVs alone were insufficient to kill leukemic cells, they did not show the promoting effect on leukemic cell growth *in vitro*. Moreover, the MSC‐EVs partially induce leukemic cell apoptosis, which would be beneficial for suppression of minimal residual disease, in particular during treatment with HSCT. In our finding, the study with NB4 and K562 enlightens the influence of MSC‐EV on some types of leukemic cell lines. The molecular mechanism causing cell apoptosis seemed to be different between both cell lines. Therefore, further studies involving more advanced techniques, testing with other leukemic cell lines, as well as the primary leukemic cells are highly needed. In addition, the combination of MSC‐EVs with known leukemia chemotherapeutic drugs is interesting in order to investigate the beneficial roles of MSC‐EVs as synergistic effects with the former treatments. Moreover, as in a review by Bailey et al [27], the result from an *in vivo* model might be different from the *in vitro* study, especially when using unmodified MSC‐EVs. Thus, the functional test the *in vivo* model is required before clinical application.

## Conflict of interest

The authors declare no conflicts of interest.

## Author contributions

AS and JP designed the study. JP performed the experiments and analyzed the data. TT collected the BM tissue. PO, DT, and SB participated in the interpretation of the data. AS, JP, and WK wrote the article.

## Supporting information


**Fig. S1.** Chondrogenic differentiation potential of BMMSCs.

## Data Availability

All data generated in this study are included in the published article.
