# Supplementary material for: Bone marrow‐mesenchymal stem cell‐derived extracellular vesicles affect proliferation and apoptosis of leukemia cells in vitro
Source: FEBS Open Bio. 2021 Dec 24;12(2):470–9. doi: 10.1002/2211-5463.13352 (PMC8804606; doi:10.1002/2211-5463.13352)
Supplement: Supplementary file 1 — Fig. S1. Chondrogenic differentiation potential of BMMSCs. [file FEB4-12-470-s001.docx]

**Supplementary data**

**
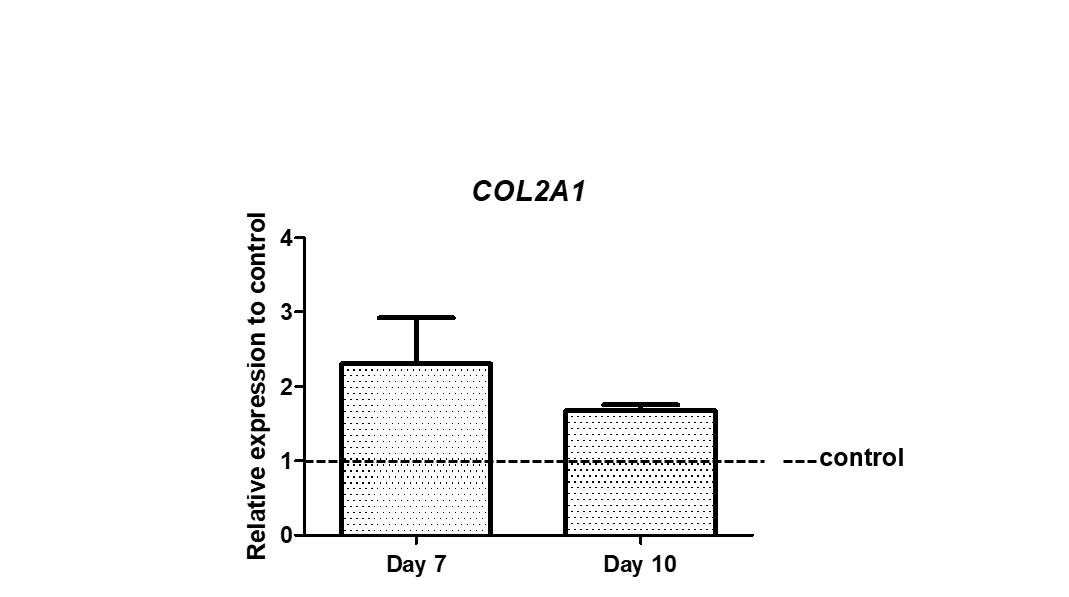
**

**Supplementary figure 1.** Chondrogenic differentiation potential of BMMSCs. The cells were cultured in chondrogenic differentiation medium which comprises of high glucose DMEM, 10% FBS, 100 nM dexamethasone, 35 µg/ml ascorbic acid, 1X Insulin-Transferrin-Selenium (ITS), 10 ng/ml transforming growth factor-beta 3 (TGF-β3), and 1% penicillin/streptomycin for 10 days. At day 7 and 10 of differentiation, the expression of *COL2A1* gene which is a specific gene expressed by chondrogenic cells was examined. Level of mRNA expression was normalized to GAPDH, housekeeping gene and presented as relative expression compared to control (mean ± SD). Primers for *COL2A1*: forward 5’-CCTGGTCTTGGTGGAAACTT-3’, reverse 5’-CAGAGACACCAGGTTCACCA-3’.
